# Dietary Plant-Origin Bio-Active Compounds, Intestinal Functionality, and Microbiome

**DOI:** 10.3390/nu12113223

**Published:** 2020-10-22

**Authors:** Elad Tako

**Affiliations:** Department of Food Science, Cornell University, Stocking Hall, Ithaca, NY 14853-7201, USA; et79@cornell.edu

**Keywords:** plant origin, bio-active compounds, intestine, microbiome

## Abstract

In recent years, plant-origin bio-active compounds in foods (staple crops, fruit, vegetables, and others) have been gaining interest, and processes to consider them for public health recommendations are being presented and discussed in the literature. However, at times, it may be challenging to demonstrate causality, and there often is not a single compound–single effect relationship. Furthermore, it was suggested that health benefits may be due to metabolites produced by the host or gut microbiome rather than the food constituent per se. Over the years, compounds that were investigated were shown to increase gut microbial diversity, improve endothelial function, improve cognitive function, reduce bone loss, and many others. More recently, an additional and significant body of evidence further demonstrated the nutritional role and potential effects that plant-origin bio-active compounds might have on intestinal functionality (specifically the duodenal brush border membrane, morphology, and the abundance of health-promoting bacterial populations). Hence, the special issue “Dietary Plant-Origin Bio-Active Compounds, Intestinal Functionality, and Microbiome” comprises 11 peer-reviewed papers on the most recent evidence regarding the potential dietary intake and effects of plant-origin bio-active compounds on intestinal functionality, primarily in the context of brush border functional proteins (enzymes and transporters), mineral (and other nutrients) dietary bioavailability, and the intestinal microbiome. Original contributions and literature reviews further demonstrated the potential dietary relevance that plant bio-active compounds hold in human health and development. This editorial provides a brief and concise overview that addresses and summarizes the content of the *Dietary Plant-Origin Bio-Active Compounds, Intestinal Functionality, and Microbiome* special issue.

The purpose of the current special issue is to further expand and add research knowledge of the vital role dietary plant-origin bio-active compounds hold in various nutrition-related physiological and metabolic pathways. In addition, the purpose is to further contribute to the knowledge regarding the relationship between plant-origin bio-active compounds, the intestinal morphology and functionality, and potential effects on the intestinal microbiome.

Plant-based diets contain a plethora of metabolites that may impact on health and disease prevention. Most are focused on the potential bioactivity and nutritional relevance of several classes of phytochemicals, such as polyphenols, flavonoids, carotenoids, phyto-estrogens, and frucrooligo-saccharides [1]. These compounds are found in fruit, vegetables, and herbs [2]. Daily intakes of some of these compounds may exceed 100 mg. Moreover, intestinal bacterial activity may transform complex compounds such as anthocyanins, procyanidins, and isoflavones into simple phenolic metabolites [3]. The colon is thus a rich source of potentially active phenolic acids that may impact both locally and systemically on gut health. Furthermore, non-digestible fiber (prebiotics) are dietary substrates that selectively promote proliferation and/or activity of health-promoting bacterial populations in the colon [4]. Prebiotics, such as inulin, raffinose, and stachyose, have a proven ability to promote the abundance of intestinal bacterial populations, which may provide additional health benefits to the host [5,6,7,8,9,10]. Furthermore, various pulse seed soluble (fiber) extracts are responsible for improving gastrointestinal motility, intestinal functionality and morphology, and mineral absorption [9,11]. Studies have indicated that the consumption of seed-origin soluble extracts can up-regulate the expression of brush border membrane (BBM) proteins that contribute for digestion and absorption of nutrients. The soluble extracts can positively affect intestinal health by increasing the mucus production, goblet cells number/diameter, villus surface area, and crypt depth [9,10]. These functional and morphological effects appear to occur due to the increased motility of the digestive tract, leading to hyperplasia and/or hypertrophy of muscle cells. Plant-origin soluble extracts may act, directly or indirectly, as a factor that increases mineral solubility and, therefore, dietary bioavailability. This occurs due to fiber fermentation and bacterial production of short chain fatty acids (SCFA) that reduces intestinal pH, inhibits the growth of potentially pathogenic bacterial population and increases the solubility and, therefore, absorption of minerals. The SCFA can increase the proliferation of epithelial cells, which, in return, increase the absorptive surface area, which contributes to the absorption of nutrients [12,13]. Several phenolic acids and other phytochemicals affect the expression and activity of enzymes involved in the production of inflammatory mediators of pathways thought to be important in the development of gut disorders including colon cancer. However, it is still unclear as to which of these compounds are beneficial to gut health. Hence, the aim of the current special issue is to further explore the interactions between dietary plant-origin bio-active compounds, their potential effects on the intestinal bacterial populations, and overall intestinal functionality and gut health.

This monograph, based on a special issue of Nutrients, contains 11 manuscripts—1 review and 10 original publications—that reflect the wide spectrum of currently conducted research in the field of dietary plant-origin bio-active compounds, intestinal functionality, and microbiome. The manuscripts in this special issue collection include contributors and researchers from multiple countries, including USA, Canada, Australia, Brazil, Poland, Finland, Belgium, Netherlands, and Spain. The presented manuscripts cover a wide variety and range of topics in the field of dietary plant-origin bio-active compounds, intestinal functionality, and microbiome, with emphasis on diet and intestinal well-being and compositions of fecal microbiota and short chain fatty acids in oat and by using subjects with celiac disease or gluten sensitivity [14]. The demonstration of low phytate peas (Pisum sativum L.)-based diets improve iron status, gut microbiome, and brush border membrane functionality in vivo (*Gallus gallus*) [15]. The presentation of novel non-digestible, carrot-derived polysaccharide (cRG-I) and how it selectively modulates the human gut microbiota while promoting gut barrier integrity (an integrated in vitro approach) [16]. The in vitro evaluation of prebiotic properties of a commercial artichoke inflorescence extract revealed bifidogenic effects [17]. The discussion of possible protective effects of traumatic acid (TA) on the cancerous effect of mesotrione [18]. Is Acrylamide as a harmful as we think? A new look at the impact of Acrylamide on the viability of beneficial intestinal bacteria of the genus *Lactobacillus* [19] The biological activity of new cichoric acid–metal complexes in bacterial strains, yeast-like fungi, and human cell cultures in vitro [20]. The presentation of how soluble extracts from chia seed (*Salvia hispanica* L.) affect brush border membrane functionality, morphology and intestinal bacterial populations in vivo (*Gallus gallus*) [21]. The fructose consumption by adult rats exposed to dexamethasone in utero changes the phenotype of intestinal epithelial cells and exacerbates intestinal gluconeogenesis [22]. Alterations in the intestinal morphology, gut microbiota, and trace mineral status following intra-amniotic administration (Gallus gallus) of teff (Eragrostis tef) seed extracts [23]. Non-dairy fermented beverages as potential carriers to ensure probiotics, prebiotics, and bio-active compounds arrival to the gut and their health benefits [24]. These wide spectra of topics further demonstrate the importance and relevance of dietary plant-origin bio-active compounds, and their effects on intestinal functionality and microbiome.

This special issue and collection of manuscripts is a useful summary of progress in various areas related to dietary plant-origin bio-active compounds, intestinal functionality, and microbiome. It also points to additional research needs, including recommendations for future research in the field, and to better understand the dietary role that dietary plant-origin bio-active compounds hold and regarding human nutrition and overall health.
